# Retreatment of failed revascularization/revitalization of immature permanent tooth – A case report

**DOI:** 10.4317/jced.53745

**Published:** 2018-02-01

**Authors:** Radovan Žižka, Jiří Šedý, Iva Voborná

**Affiliations:** 1 Institute of Oral Sciences and Dentistry, Faculty of Medicine, Palacky University, Olomouc, Czech Republic; 2 Department of Anatomy, Faculty of Medicine and Dentistry, Palacky University, Olomouc, Czech Republic

## Abstract

The aim of this article is to describe the clinical case of the non-surgical retreatment of a failed revascularization/revitalization treatment (RRT). The retreatment was preceded by two sessions including an interim medication with calcium hydroxide and a modified irrigation protocol containing a highly-concentrated sodium hypochlorite and prolonged usage of ethylenediaminetetraacetic acid (EDTA) to reverse unacceptable side effects of hypochlorite. During a 15- month follow-up, the healing of the periapical lesion, increased thickening of root canal walls and maturation of root was apparent. The repeated RRT represents a possible treatment modality for s failed RRT.

** Key words:**Retreatment, revascularization, revitalization, maturogenesis, immature tooth.

## Introduction

The treatment of immature permanent teeth with necrotic pulp presents many challenges that place demands on dental specialists and compromise the prognosis of teeth. Disinfection of the root canals introduces several problems such as cleaning and shaping large canals with open apices, the obturation and potential root fractures due to thin root canal walls (1). During last decade, a set of a novel techniques were proposed to overcome calcium hydroxide apexification which has been the gold standard for several decades. These techniques are called cell-free regenerative endodontic procedures (REP) in summary. They are also known as revascularizaton, revitalization or maturogenesis. The first case report of a modern regenerative procedure was reported in 2001 by S. I. Iwaya (2). Since then many case reports and case series have been published. At first, the great emphasis was on root canal disinfection considered necessary for pulp tissue regeneration (1). To fulfil the requirement of a bacteria-free root canal system, multiple visits with temporary antibiotic intracanal medication and irrigation with high concentration sodium hypochlorite were prearranged. Since then several laboratory studies have focused on root canal disinfection since they improved the quality and amount of new tissue. This led to the development of a current treatment protocol, recommended by the American Association of Endodontists (AAE). It consists of short-time usage of low concentration of sodium hypochlorite, which reduces the proteolytic effect on stem cells and dentin, especially on the dentinal matrix with incorporated growth factors. However, this leads to low action against an extracellular matrix of biofilm (3). The negative effect of sodium hypochlorite on dentin is reduced by prolonged usage of EDTA (4). Residual bacterial infection is decreased by interim medication with a low concentration of triple antibiotic paste or calcium hydroxide (5). Experiments have shown that residual bacteria persisted in non-instrumented recesses of root canals and isthmuses which makes it impossible to eliminate them by sodium hypochlorite or by two-visit treatment protocols, thereby theoretically reaching the goal of achieving a microbe-free canal by the root canal treatment that is currently unattainable (6). If the RRT is unsuccessful, it could be due to insufficient disinfection of the root canal system although evidence in the literature is meagre. If RRT fails to occur, there is no guideline for further treatment. It is assumed that conventional treatment should be performed. This case report presents an unusual instance of RRT after previous RRT failed.

## Case Report

**Figure 1:**
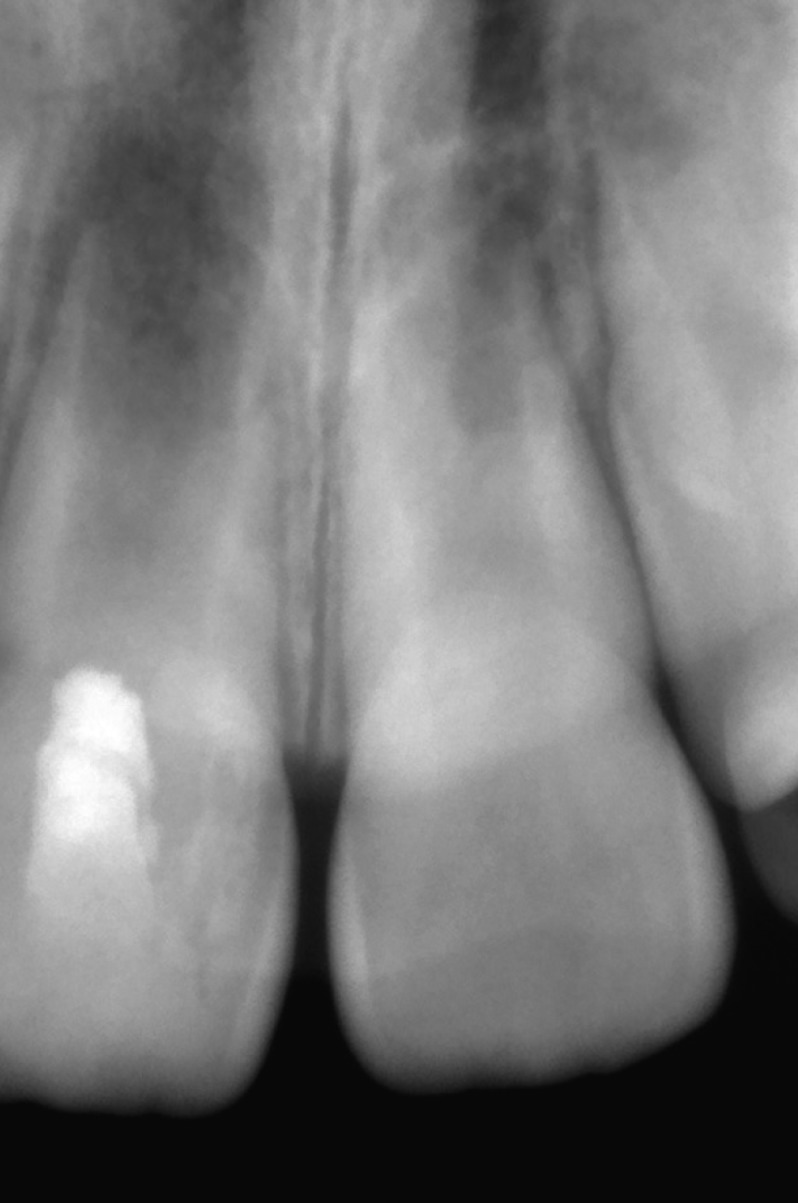
Diagnostic intraoral x-ray of the central incisor with failed revascularization treatment. As visible, mesial pulp horn was not included to access cavity design.

**Figure 2:**
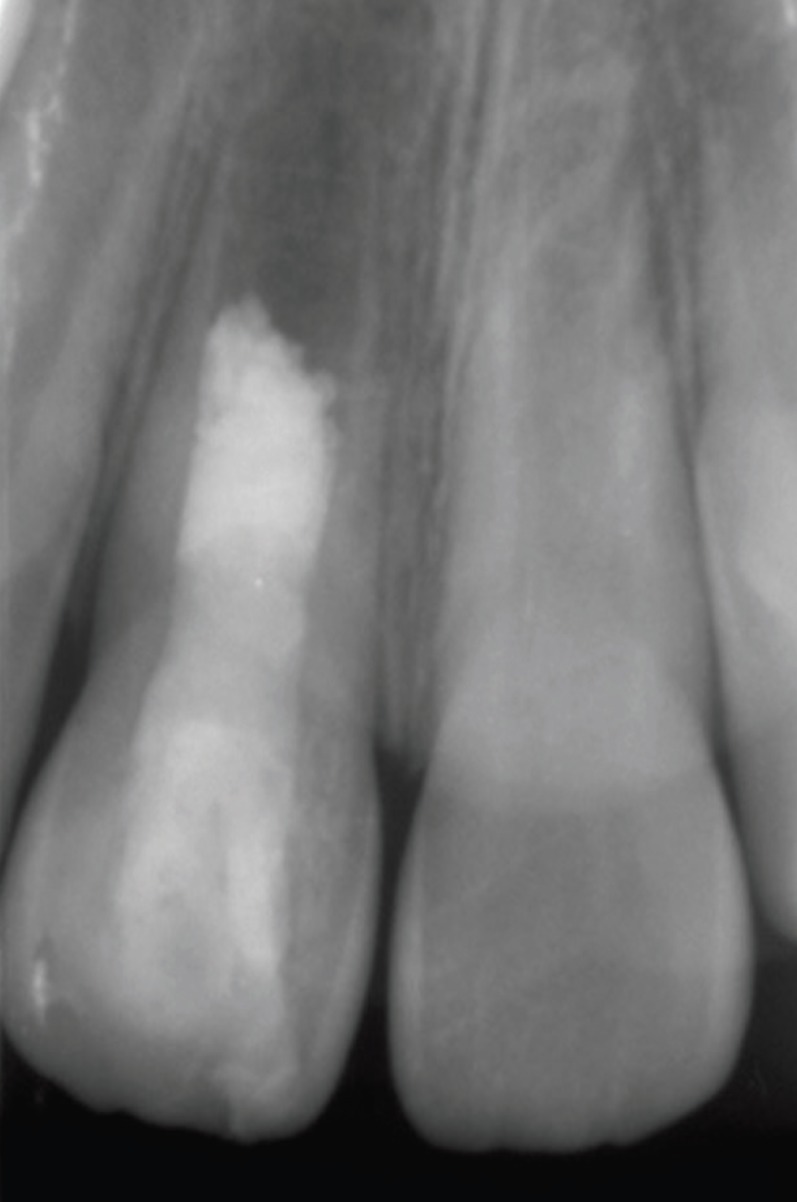
Postoperative -ray after non-surgical retreatment of failed revascularization/revitalization of the immature first central incisor.

**Figure 3:**
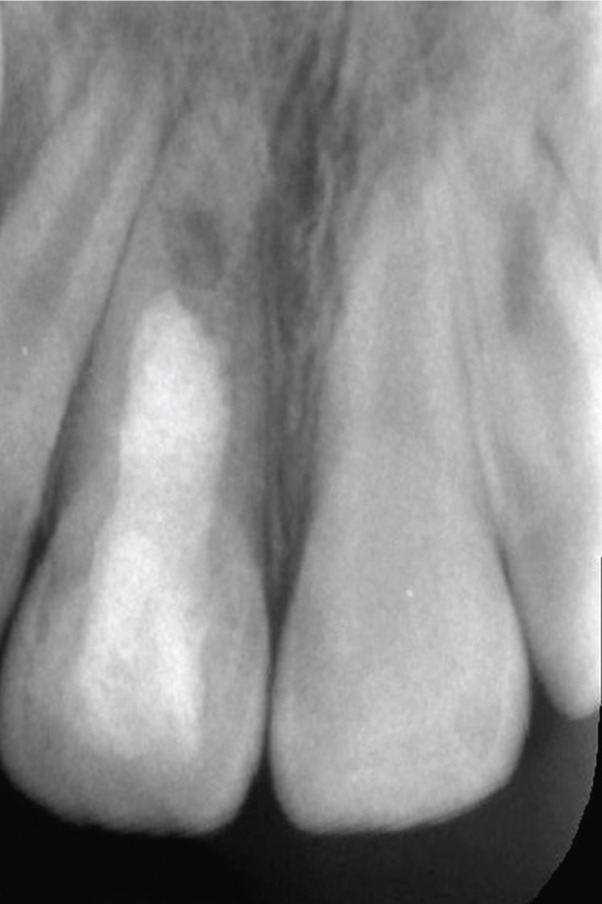
Follow-up at 15 months. Periapical lesion diminished. Maturation of root apex and increase in root canal wall thickness are apparent.

A 7-year-old female patient was referred to our department for the evaluation and treatment of a maxillary anterior swelling associated with her permanent maxillary right incisor. The cervical area of the tooth had a slightly greyish discoloration due to presence of mineral trioxide aggregate (MTA), and an access cavity was adhesively restored. Palpation was mildly painful but responses to percussing and probing the pocket depth were all within normal limits. A diagnostic x-ray from a referral dentist showed that the tooth was in the second stage of root development and revealed a periapical lesion and radiopaque material in the pulp chamber. The access cavity was inadequate because the mesial pulp horn was not included in the access cavity design (Fig. 1). The tooth was diagnosed as acute exacerbation of chronic apical periodontitis with maxillary abscess. The dental history disclosed that the patient was treated two weeks earlier when the RRT was performed according to AAE’s guidelines. The dental history also disclosed the patient had suffered dental trauma nearly 4 months earlier, sustaining a subluxation of her permanent maxillary right incisor. After discussion with the patient´s parents about possible treatment modalities and the signing of informed consent forms, the RRT was repeated. During the first appointment, local anesthesia 4% articaine with 1:200,000 epinephrine (Supracain 4%; Zentiva a.s., Prague, Czech Republic) and a rubber dam were applied and the access cavity was redesigned. Then the orientation working length was established by an electronic apex locator and verified by a measuring x-ray. The root canal system was irrigated for 20 minutes by 5% sodium hypochlorite, which was ultrasonically activated for 5 minutes, rinsed with saline and dried. Calcium hydroxide was applied to the coronal third and access and covered with sterile Teflon tape, thereby temporarily restoring the access cavity with glass ionomer cement. Then a puncture released pus from abscess. After 4 weeks the patient was fully asymptomatic and the second part of the RRT was initiated. In local anesthesia, 4% mepivacaine (Mepivastesin; 3M ESPE, St Paul, MN), a rubber dam was applied and a temporary glass ionomer restoration with Teflon tape was removed. The root canal system was irrigated for 10 minutes with 5% sodium hypochlorite and subsequently with 17% EDTA for 20 minutes in order to compensate for the unacceptable effect of sodium hypochlorite on the dentin. Before induction of bleeding the whole root canal was rinsed with 10 mL of sterile saline. Afterwards, bleeding from apical papilla was induced with a sterile ISO 25 K-file, with the tip bent by 30°. A blood clot was stabilized within 3 minutes by oxidized cellulosis (Traumacel Newdent, Bioster, Czech Republic). White MTA (ProRoot MTA; Dentsply Tulsa Dental, Johnson City, TN) was mixed with sterile water and applied over the stabilized blood clot together with sterile paper points. The access cavity was adhesively restored with a composite filling. Despite an effort to place MTA just 1 mm below the cement-enamel junction, it was placed near the middle of the root canal system, as visible on postoperative X-ray (Fig. 2). During the follow-up at intervals of 3, 5, 9, 12 and 15 months, the patient was completely asymptomatic. Compared with her adjacent and contralateral teeth, her central right incisor was within normal limits regarding percussion, palpation and pocket depths. It was not responsive to heat or cold pulp vitality tests. Radiographic evaluation showed a reduction of the size of periapical radiolucency and further development of tooth type I, according to Chen *et al.* (7) (Fig. 3). Interestingly the length of the treated right central incisor is larger than a healthy non-treated left central incisor. Equally interesting is the apposition of mineralized tissue in contact with MTA.

## Discussion

Many complications of regenerative endodontic procedures have been described, such as discoloration of the clinical crown, inability to induce bleeding from apical papilla or the collapse of MTA into the blood clot (8). Scientific reports about the failure of regenerative endodontic therapy are rare in the literature (9). These reports are concerned with a limited deposition of mineralized tissue or development of the tooth rather than to recurrent bacterial infection (7,10). If RRT is not successful, the recommended procedure is extraction or conventional root canal treatment with MTA apexification (10,11). The most probable cause of RRT failure is residual bacterial infection (11). In this specific case the most probable source of bacterial infection was infected pulp tissue because of improper access cavity design but we cannot rule out severe bacterial infection of the root canal system. We were concerned about possible severe intracanal infection, so used 5% hypochlorite for a prolonged period of time with thorough ultrasonic activation. In the literature an unacceptable effect of highly concentrated hypochlorite on dentin and dental pulp stem cells is well described in regenerative endodontics (12). This undesirable action can be successfully reversed by prolonged irrigation with EDTA (4) as was done in this case report. We must point out that in this specific case of RRT the chief aim was not the protection of stem cells and growth factors on the dentine, but rather the elimination of bacteria and the preparation of a suitable environment for further treatment. The unintentional placement of MTA below the cement-enamel junction has not vitiated a favourable outcome of the RRT. On the one hand, there was no gain of mineralized tissue in the cervical third of root, so the fracture resistance of tooth was not improved. However, we can assume that such a deep placement of MTA reduced root canal space which could help regenerate or repair the defect. This assumption is in agreement with the concept of “critical size of defect” (13,14). Critical size defect for wound-healing studies on animals , without introducing any supportive approaches, reveals the defected area will not regenerate naturally during its continuance (14). The critical size defect for dental pulp regeneration has not yet been studied. In the literature there is evidence that improper placement of MTA could lead to formation of mineralized tissue as an apical barrier (15). In the revascularization retreatment, dentists should be aware of the risk of the previous damage to the apical papilla and Hertwig´s epithelial sheath by prolonged inflammation or trauma during the disinfection protocol or induction of bleeding. It is correct to assume that revascularization/revitalization retreatment will have a lower success rate than the primary attempt.
